# Doing Socially Responsible Science in the Age of Selfies and Immediacy

**DOI:** 10.1523/ENEURO.0114-22.2022

**Published:** 2022-03-28

**Authors:** Christophe Bernard

Responsible science has three components: doing science, the validity of the discoveries themselves, and the consequences of these discoveries. These three components are nondissociable, because science does not exist by and for itself: it exists within a societal context. Society and Science always interact with each other. Doing science has direct societal consequences, which can be positive, including novel therapeutic solutions and general advancement of knowledge, and negative, including using planet resources, producing waste, and contributing to global warming (with travel, for example). I shall not develop the latter components here; I shall develop the validity of the discoveries and their consequences in the present context of the immediacy of information and “selfie” science.

An idealistic and naive depiction of a scientist is someone concerned only with the internal content of their scientific work and not with their external repercussions. A scientist is but one part of the complex organism that is human society. Except for the now rare cases when a scientist is wealthy, a scientist cannot do any work without support from society. Since state funding comes from taxes, any money given to science is money that taxpayers cannot spend for themselves. As scientists, the least we can do is to provide feedback to society and convince it that the money spent will provide for the benefit of all. This is the second level of socially responsible science: our production must be valid. After all, if a business makes deficient products, it will not survive long. What would happen, were taxpayers convinced that science is not producing what is being promised?

The second level of responsibility to consider is when the results produced by the scientist can affect the society. It can be for the better good, as designing vaccines against viruses. Or it could have deleterious consequences. Leó Szilárd and Enrico Fermi, among others, made atomic fission possible, leading to nuclear bombs dropped on Hiroshima and Nagasaki. The knowledge of the potential destructive power of the discovery did not prevent them from continuing their research. It is tempting to say that scientists should not continue along a path such as this if they foresee negative impacts, or at the very least oppose the misuse of their work. This is also a naive statement. The state of society at any given time point imposes constraints on scientists, voluntarily or not. Pre-World War II Germany and Italy drove Leó Szilárd and Enrico Fermi to seek refuge in the United States, with the intuition that given the state of knowledge in nuclear physics at that time, Germany and USSR would also try to build fission bombs. Who is then responsible of Hiroshima and Nagasaki? Leó Szilárd wrote the letter signed by Albert Einstein, which practically launched the Manhattan project. But in 1945, the same Leó Szilárd drafted the petition advocating to warn Japan about a possible use of a fission bomb. Instead, the Interim Committee decided for bombing Japanese cities without warning. Who is socially (and morally) accountable? The results, the A-bomb, and the Cold War, are as much the fruit of a chain of scientific discoveries, scientists competing against each other to be the first to do controlled fission, as the evolution of society in time.

This leads to the “moral blindness” surrounding some experiments. I shall not dwell on that, as “moral” is time-, society-, and person-dependent. Claude Bernard argued that vivisection was justified, even when causing suffering to animals, if it was done to ultimately help humans. What was morally acceptable before is not anymore. The morality of some “scientists” may not be that of their society. The United States Army commissioned Harold Hodge to investigate what radioactive contamination was doing to humans following plutonium injections in unaware individuals. The Tuskegee Syphilis Study was only stopped in 1972, and just because of a leak to the press. Depending on financial aspects and personal beliefs, some groups did (and still do) experiments which would be deemed unacceptable by most (but again based on the moral state of the society we live in).

The latter examples are one extreme of the spectrum, where the application of discoveries can directly lead to the suffering and death of many. Before the generalization of Internet use, science was not accessible to everyone. It entered our general society only when journalists would select a particular topic to cover. In other words, scientists were mostly accountable to their own community and funding agencies. Things changed progressively as scientists started to communicate their results to a much broader audience. The development of the Internet and social media, and more generally the immediacy of access to information, considerably accelerated the diffusion of scientific results as well as of misinformation to society. As we switch to “science in the age of selfies” (Geman and Geman, 2016), one could think that exposing ourselves to the public eye would be beneficial to both science and society. Did such immediacy produce self-regulation and socially responsible science?

How can we define responsible science in the present state of the society? Responsibilities are shared by the multiple actors. They include the scientists themselves, the journals that evaluate and publish their results, and the journalists. Let us start with the source: scientists, who can be separated into those with and without a specific agenda. The satisfaction of one’s ego being common to all, I do not consider it as a specific agenda even if it is the main engine of our actions. Likewise, I do not consider as a specific agenda, that we all have to pursue our work and obtain funding.

1) The scientist without agenda. Communication through social networks has become an essential tool to many scientists not only to have their work known by other scientists but also to force their way to and justify their position at the top of the pyramid (to obtain grants, prizes, positions, etc.). In addition, scientific journals use various metrics to show the impact of research on Twitter, blogs, news channels, etc.; parameters that may be considered by grant and recruiting committees. Thus, it is tempting for scientists to overinterpret or oversell their results to make the buzz. This is reflected in flashy paper titles or conclusions (e.g., targeting protein X prevents Alzheimer’s disease). Although it is important to communicate to the public what scientists do, it is equally important not to give false hopes. This is best exemplified with a novel -omics field: promisomics (Gomez-Marin, 2021), the tendency to overpromise (and underdeliver). Since research money is money taken from taxpayers, the latter want to hear success stories, rather than incremental steps. According to the titles in top-tier journals over the past decades, epilepsy, Alzheimer’s disease, depression, etc. should have been “cured” a long time ago. This puts the field in a very uncomfortable position. Given the present lack of success in treating major neurological disorders, and the lack of real scientific breakthrough as consistently claimed in high-tier journals, the public may not be so supportive for research in these fields. This could become a political campaign argument: cutting national research budgets to increase the amount of money that remains in the taxpayer’s wallet.

But how can we be honest about the real impact of our findings? Just a single brick in the wall, the wall representing the community-building of scientific knowledge, will not satisfy top-tier journals. If we don’t play the game of inflating the importance of our results, we may not survive (not getting grants or jobs). So many of us overdo it; we oversell or overpromise, falling into the promisomics pitfall. Most often our “salesmanship” ends up doing nothing but generating false hopes and leaving the public with the impression that we are making big strides while we are just making small steps. As scientists, we bear a large responsibility for striking a better balance between hype and substance. The game is not imposed on us by others, we are its primary source. We should stop and think more about what we are doing. But can we? We should be humbler with respect to what our results really mean. Relying on self-regulation is a pious wish because in an environment with limited resources any organism needs to fight to survive; this is the number one rule of biology. In such a context, anything that can give us an edge against other scientists is worth using. We all know that to be published in top-tier journals, we will need a flashy and punchy title that will attract the crowds (and the editors of these journals). If we are not striving to promote our work in the (social) media, while others do, we decrease our chances. Since we cannot regulate ourselves, who can? I am not sure that one solution would be to leave the responsibility of communication to universities or funding agencies, as they already compete against each other for prestige (e.g., the Shanghai ranking), because in the end it is again a question of money at their own level. Except for changing the way society works, I fail to identify solutions. Feel free to discuss this issue (and the following ones) on our blog (https://blog.eneuro.org/).

2) The scientist with an agenda. This can take many forms. A scientist, or a community of scientists, may have proposed a theory that they wish to forcibly validate. Alzheimer’s disease and the failure of recent clinical trials provide a good illustration (Herrup, 2022). This state emerges from the science field itself. It can start with a few vocal scientists, a simple but appealing idea etc. This is how dogma emerges. For dogma to persist, one needs constant fueling the field with results in line with the dogma. Those aligned with the dogma are more likely to get funded (and publish more papers supporting the dogma) and obtain jobs. Fighting dogmas has always been difficult throughout history because it is far more comfortable to rely on principles that we believe are stable and because, if you oppose it, you are more likely to be stigmatized or kicked out of the system. One solution is to train students to have a critical eye as early as possible in their curriculum, in particular by teaching them to spot fallacies (Bernard, 2020). But knowing about problems does not necessarily lead to corrective actions. I remember that when I started as a PhD student, there were very few women scientists, and nearly none as department heads. We, students, were saying: this will not occur under our watch when we get to the decision-making level. Well, the same argument has been used by multiple generations of young scientists, and the lack of gender parity is still appalling. Pessimistic? No, factual.

Another variation of this problem is a dogmatic view on a subject that one wishes to force into the public domain. For some reason, a group of scientists despises people with long hair. One of them manages to publish a paper where it is shown that people with long hair have a lower IQ, are lazy and benefit from society without giving anything in return. This can spread like wildfire in social media by all citizens who happen to despise people with long hair. This could result in a stigmatization of people with long hair, generating a lot of suffering in their community.

Since we are supposed to be “thinkers,” scientific debate should resolve such issues. This is not the case. Individuals are likely to vocally support what they believe in, or what they have been trained to believe, no matter what. Again, education may be the way forward. It is quite amazing to see nowadays how fast and deep science percolates through all the layers of society. Yet, there is a major discrepancy with what lay persons can consider with a critical eye because we are not educated enough not only in different science fields but also in how to evaluate science itself.

After the scientists, the other component to consider are the scientific journals. Let us start with the simple case for which procedures are in place. As mentioned, research money is limited, and we compete to obtain a share of it. This tension too easily leads to misconduct, including fabrication, falsification, plagiarism, and conflict of interest, to publish in top-tier journals, which is often a prerequisite to obtaining grants or jobs. Journals have a large arsenal of tools to assess possible misconduct. The task can be cumbersome, but an objective decision like retraction or correction can be reached, even after publication, if a paper is faulty.

Academic publishing is a profitable business. This spawned the arrival of a flurry of predatory and fake journals in which you can publish anything if you pay the fees. Perhaps there is a paper out there claiming that the only answer to COVID-19 is “42.” These “journals” appear to have a scientific veneer, and if a scientist has an agenda, it is easy to fulfill it, even more so if the message is relayed by the media.

Most high-tier journals are for profit and not handled by active neuroscientists. Making money is at the core of their operational strategy. This means that their articles must achieve constant public exposure to generate more prestige and hence more revenue, generating a virtuous (from their viewpoint) cycle. This explains the flashy titles aimed at selling, not informing. Sometimes, it borders on the predatory. Perhaps it is not done on purpose; it could be just an emergent property of the larger system (doing research + evaluation by peers + publication) that evolved for the past 150 years. My take is that we all share responsibility.

Although journals should have procedures in place to prevent damages, bad things can happen. Many factors can explain why bad papers have been and are still being published: we are not careful enough as editors or reviewing editors, the message aligns with common views, it appears like a great result, etc. Numerous stories too good to be true or too groundbreaking to be true have been published and retracted. However, because of the publicity generated, the damage can be enormous. Perhaps the best example is the paper published in The Lancet linking autism to MMR vaccines, which was retracted only 12 years later. It is still generating a lot of damage and misinformation.

Does the publisher bear responsibility for the deleterious effects that the paper had in terms of conspiracy theories, unvaccinated children, etc.? It is easy for a journal to wash its hands, blaming the reviewers who did not do a proper job and accepted the paper. This argument is only partly valid. An editor, in particular in a high-tier, for-profit journal, may have as part of their agenda generating the buzz in the media and among scientists. They may also find the topic to be particularly appealing and so select reviewers who may be predisposed to like the story (note that the reverse is true, hatchet reviewers can be selected with the purpose to reject a paper). Editors may also disregard negative reviews because they want the story to be published (this happened to me in several instances when reviewing for high-tier journals). To decrease the occurrence of such bad cases, the solution is straightforward: the review process should be explicit and transparent. If reviewers and editors become accountable for what is published, providing the reviews and the name of the actors, we may end up with less flashy or deleterious and more accurate science.

Yet, there is a question for which I do not have an answer: as a journal should we scrutinize every paper which could be potentially damaging? In a society where immediacy is the rule, a Tweet can generate a Capitol offense (as spelled). If the study showing that people with long hair have a lower IQ is based on solid results, should we refrain to publish it because of the potential backlash? Where does social responsibility start? In contrast to what we measure in neuroscience, societal values are society-, community-, and individual-dependent. Small groups of activists can sometimes be the most vocal. Since we evolved to selfie science and immediacy, without taking the time to pause and ponder, how can a journal deal with results that *may* be seen as offensive by some. Should we censor ourselves?

The media are also key actors. Since there are many articles on this topic, I will not develop it. Naively, before the Internet, social media, and the immediacy of information, journalists could take time to work on a subject. The primacy now is to be the first to relay and publish information, and anyone can claim to be “specialist” with a justification to relay scientific results. The society should also consider the accountability of those relaying damaging or inaccurate information.

In conclusion, socially responsible science is a complex question where all actors can bear some responsibility. The question is not novel, it was already raised in 1935 (Science and Social Responsibility, 1935), and perhaps even before that. Yet while it may be déjà vu, this is like the gender parity issue. It needs to be highlighted again and again. How to do socially responsible science? I argue that there cannot be a final answer, because “responsibility,” “society,” and even “science” itself are labile concepts that change in time and space. Society is a complex system, like the brain, where all components interact with each other. A weak argument for scientists to use would be let us wait for society to change, not our business. If we are part of the problem, are we not morally bound to act now? We must consider not just what is our best strategic move, but also what is the responsible thing to do. Such humanistic views are easy to support when we are students, but they are quickly set aside when we enter the competition, when it becomes a question of survival. Education is key, but it should not stop at students. Socially responsible science could be part of a training program that every scientist should attend every X years. Even if we will always be driven by our ego, we can learn how to control it to favor substance instead of hype. Solutions are easier to implement at the journal level. At *eNeuro*, we believe that all the editors handling our papers should be active scientists, and the review process should be transparent (i.e., publishing the reviews, the names of reviewers, and reviewing editor). Let us favor the “maximize knowledge and education” principle against the “maximize money” principle.
